# 1,25-Dihydroxyvitamin D_3_ (1,25(OH)_2_D_3_) Signaling Capacity and the Epithelial-Mesenchymal Transition in Non-Small Cell Lung Cancer (NSCLC): Implications for Use of 1,25(OH)_2_D_3_ in NSCLC Treatment

**DOI:** 10.3390/cancers5041504

**Published:** 2013-11-08

**Authors:** Santosh Kumar Upadhyay, Alissa Verone, Suzanne Shoemaker, Maochun Qin, Song Liu, Moray Campbell, Pamela A. Hershberger

**Affiliations:** 1 Department of Pharmacology and Therapeutics, Roswell Park Cancer Institute, Elm and Carlton Streets, Buffalo, NY 14263, USA; E-Mails: upadhyaysk97@gmail.com (S.K.U.); alissa.verone@roswellpark.org (A.V.); suzanne.shoemaker@roswellpark.org (S.S.); moray.campbell@roswellpark.org (M.C.); 2 Department of Biostatistics and Bioinformatics, Roswell Park Cancer Institute; Elm and Carlton Streets, Buffalo, NY 14263, USA; E-Mails: maochun.qin@roswellpark.org (M.Q.); song.liu@roswellpark.org (S.L.)

**Keywords:** epithelial mesenchymal transition, vitamin D, 1,25-dihydroxyvitamin D_3_, lung cancer, TGFβ

## Abstract

1,25-dihydroxyvitamin D_3_ (1,25(OH)_2_D_3_) exerts anti-proliferative activity by binding to the vitamin D receptor (VDR) and regulating gene expression. We previously reported that non-small cell lung cancer (NSCLC) cells which harbor epidermal growth factor receptor (*EGFR*) mutations display elevated *VDR* expression (VDR^high^) and are vitamin D-sensitive. Conversely, those with *K-ras* mutations are VDR^low^ and vitamin D-refractory. Because *EGFR* mutations are found predominately in NSCLC cells with an epithelial phenotype and *K-ras* mutations are more common in cells with a mesenchymal phenotype, we investigated the relationship between vitamin D signaling capacity and the epithelial mesenchymal transition (EMT). Using NSCLC cell lines and publically available lung cancer cell line microarray data, we identified a relationship between *VDR* expression, 1,25(OH)_2_D_3_ sensitivity, and EMT phenotype. Further, we discovered that 1,25(OH)_2_D_3_ induces *E-cadherin* and decreases EMT-related molecules *SNAIL*, *ZEB1*, and *vimentin* in NSCLC cells. 1,25(OH)_2_D_3_-mediated changes in gene expression are associated with a significant decrease in cell migration and maintenance of epithelial morphology. These data indicate that 1,25(OH)_2_D_3_ opposes EMT in NSCLC cells. Because EMT is associated with increased migration, invasion, and chemoresistance, our data imply that 1,25(OH)_2_D_3_ may prevent lung cancer progression in a molecularly defined subset of NSCLC patients.

## 1. Introduction

1,25-Dihydroxyvitamin D_3_ (1,25(OH)_2_D_3_), the active metabolite of vitamin D, exerts anti-cancer activities by binding to the vitamin D receptor (VDR) and modulating gene expression [1]. Historically, the anti-tumor activity of 1,25(OH)_2_D_3_ has been attributed largely to its ability to suppress cell cycle progression via the induction of cyclin dependent kinase inhibitors p21^waf1^ and p27^kip1^ [2,3,4,5,6]. However, more recent studies demonstrate that 1,25(OH)_2_D_3_ inhibits a number of additional processes critical to tumor survival and progression including angiogenesis [7,8,9], telomerase activation [10,11], and the epithelial-mesenchymal transition (EMT) [12,13,14,15].

EMT refers to a process in which cells lose expression of genes associated with an epithelial phenotype (such as E-cadherin (*CDH1*)) and acquire expression of genes associated with a mesenchymal phenotype (such as vimentin (*VIM*)). Transcription factors belonging to the SNAIL and ZEB families coordinate EMT by repressing *CDH1* and other cell junction proteins (reviewed in [16]). EMT-associated changes in gene expression are accompanied by alterations in cell morphology and behavior, such that cells which have undergone EMT acquire an elongated, spindle shape and display increased migration and invasiveness.

In lung cancer models, EMT confers resistance to both radiation and chemotherapy [17,18]. EMT also determines the therapeutic response of NSCLC cells to epidermal growth factor receptor (EGFR) tyrosine kinase inhibitors erlotinib and gefitinib. In 2005 it was discovered that NSCLC cells with wild-type *EGFR* display a range of sensitivities to erlotinib, and that sensitivity depends on whether the cells express CDH1 or VIM [19]. Consistent with these findings, *CDH1* transfection was demonstrated to be sufficient to sensitize NSCLC cells to EGFR tyrosine kinase inhibitors [20]. At the same time, microarray approaches were used to uncover the basis for the differential responsiveness of NSCLC cells to erlotinib. These also resulted in the identification of EMT as a determinant of drug sensitivity and CDH1 protein expression as a biomarker of erlotinib activity in NSCLC patients [21]. EMT also represents an important mechanism by which NSCLC cells and NSCLC patients become resistant to EGFR tyrosine kinase inhibitors during treatment [22].

To more fully characterize EMT in NSCLC and its association with drug response, Byers *et al.* recently developed and validated a 76-gene EMT signature: This signature predicts the resistance of NSCLC cells to EGFR and PI3K inhibitors and disease control in NSCLC patients receiving erlotinib [23]. Several of the NSCLC cell lines that were used in the derivation of the EMT signature were previously characterized for their sensitivity towards 1,25(OH)_2_D_3_ by us [24]. This afforded us the unique opportunity to explore the relationship between vitamin D signaling capacity and the EMT phenotype in NSCLC. Data contained in this report provide initial evidence that the EMT phenotype (as defined by the 76-gene EMT signature) discriminates between NSCLC cells that are sensitive or resistant to the growth inhibitory effects of 1,25(OH)_2_D_3_, and that the epithelial phenotype is actively supported by 1,25(OH)_2_D_3_. The implications of these findings with regard to the clinical application of vitamin D in the treatment of NSCLC are provided in the Discussion.

## 2. Results and Discussion

A 76-gene signature which classifies whether a NSCLC cell line has undergone EMT was recently described by Byers *et al.* [23]. Hierarchical clustering of 54 NSCLC cell lines based on the 76-gene signature resulted in distinct epithelial and mesencyhmal groups. Upon examining the cell lines that fell within each group, we noted a possible association between EMT phenotype and 1,25(OH)_2_D_3_ responsiveness (Table 1). Specifically, we observed that cell lines which express relatively high levels of vitamin D receptor (*VDR*) and respond to 1,25(OH)_2_D_3_ treatment (such as HCC827 and H3122 cells) have an epithelial phenotype (Table 1). Conversely, cell lines that express relatively low levels of *VDR* and are refractory to 1,25(OH)_2_D_3_ treatment (such as H23 and A549 cells) possess a mesenchymal phenotype (Table 1). A cell line was considered 1,25(OH)_2_D_3_-sensitive if treatment resulted in robust induction of the vitamin D target gene *CYP24A1* and/or growth inhibition at 10 nM 1,25(OH)_2_D_3_. These observations prompted us to examine in more detail the relationship between *VDR* expression, vitamin D sensitivity, and the EMT in NSCLC cells.

**Table 1:** Relationship between Vitamin D Signaling Pathway Integrity and EMT Phenotype in NSCLC. *VDR* and *CYP24A1* mRNA expression were measured in each cell line by qRT-PCR. *VDR* expression was measured under basal growth conditions. *CYP24A1* was measured in cells treated with either vehicle (control) or 10 nM 1,25(OH)_2_D_3_ for 8 h. *CYP24A1* induction was calculated as follows: Fold-induction = *CYP24A1* in 1,25(OH)_2_D_3_ treatment group/*CYP24A1* in control group. Clonogenic assays were used to measure growth inhibition by 1,25(OH)_2_D_3_ (10 nM), as outlined in the Experimental section. * Value was abstracted from previously published work [24,25]. The EMT phenotype was defined by Byers *et al* based on a 76-gene signature [23]. E (epithelial), M (mesenchymal), ND (not determined).

VDR Phenotype	VDR^high^				VDR^low^		
Lung Cancer Cell Line	H3122	H292	HCC827	SK-LU-1	H23	A427	A549
Normalized *VDR*	1.0	0.63	0.32	0.49	0.03	0.004	0.01
Fold-induction *CYP24A1*	3504	9791	452	27116	7.2	1.2	1.6
% Inhibition by 1,25(OH)_2_D_3_	70	73 *	82 *	30	ND	ND	4 *
EMT phenotype	E		E		M		M

### 2.1. Characterization of the Association between Vitamin D Signaling Capacity and EMT Phenotype in NSCLC Cells

**Figure 1:**
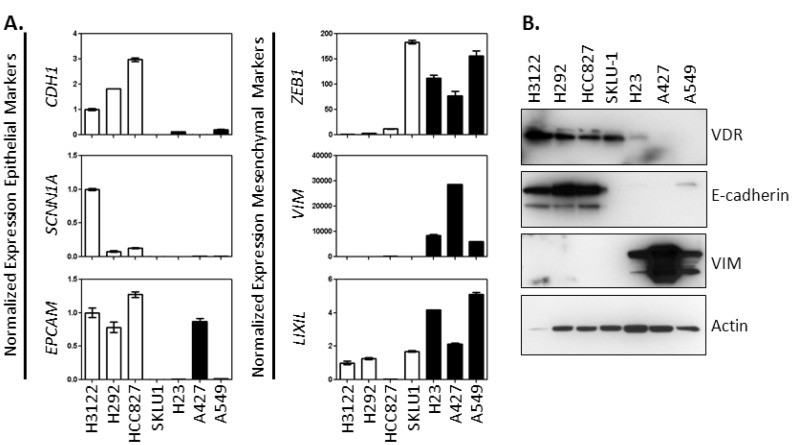
NSCLC cells that are VDR^high^ and vitamin D-sensitive preferentially express markers of an epithelial phenotype. The indicated NSCLC cell lines were grown under basal growth conditions until they achieved 50%–70% confluence. (**A**) RNA was extracted and used to prepare cDNA. One microliter of each cDNA was used in a quantitative PCR assay to measure expression of representative epithelial markers (*CDH1*, *SCNN1A*, and *EPCAM*) and mesencyhmal markers (*ZEB1*, *VIM*, and *LIX1L*). Data are the mean ± SD for triplicate determinations within a single experiment. Data were normalized to that obtained for H3122 cells. H3122 gene expression was arbitrarily assigned a value of 1.0. VDR^high^ cells are indicated with white bars. VDR^low^ cells are indicated with black bars; (**B**) Protein was extracted 24 h post-seeding of 5 × 10^6^ cells and analyzed by immunoblot for VDR, E-cadherin and VIM. Thirty micrograms of total protein was analyzed per sample.

Based on our initial observations described above, we hypothesized that EMT phenotype distinguishes between cells that express the *VDR* and are vitamin D-sensitive and those that weakly express *VDR* and are vitamin D-refractory. To test this, two approaches were taken. First, qRT-PCR was used to measure the expression of epithelial markers (*CDH1*, *SCNN1A*, and *EPCAM*) and mesenchymal markers (*ZEB1*, *VIM*, *LIX1L*) across the full set of NSCLC cell lines for which we had vitamin D sensitivity data (presented in Table 1). Markers of the epithelial phenotype were preferentially expressed in H3122, H292, and HCC827 cells that are VDR^high^ and vitamin D responsive (Figure 1). Conversely, markers of the mesenchymal phenotype were preferentially expressed in H23, A427, and A549 cells that express relatively low levels of the *VDR* and are more refractory to 1,25(OH)_2_D_3_ treatment. Using only these six genes, we could not classify SK-LU-1 cells as having a distinct epithelial or mesenchymal phenotype: SK-LU-1 cells had very low expression of all 3 epithelial markers that were tested, but they also lacked expression of *VIM*, a classical marker of the mesencyhmal phenotype. 1,25(OH)_2_D_3_ treatment of SK-LU-1 cells resulted in *CYP24A1* induction and growth suppression (Table 1). Interestingly, the magnitude of growth suppression in SK-LU-1 cells is intermediate between cells with a distinct epithelial or mesenchymal gene signature.

To confirm that the RNA-based signatures were reflected in expression of corresponding proteins, whole cell extracts were prepared from each of the cell lines and examined for expression of VDR, E-cadherin, and VIM by immunoblot. H3122, H292 and HCC827 cells that were classified as VDR^high^ and epithelial based on their RNA expression profiles displayed high expression of VDR, high expression of E-cadherin, and were VIM negative (Figure 1B). Conversely, H23, A427, and A549 cells that were classified as VDR^low^ and mesenchymal based on their RNA expression profiles displayed little to no VDR, little to no E-cadherin, and high levels of VIM. Furthermore, as predicted from the RNA data, SK-LU-1 cells expressed VDR but had undetectable levels of either E-cadherin or VIM. These data indicate high concordance between RNA and protein based EMT markers.

In a second approach, we determined the correlation between expression of *VDR* and genes included in the 76-gene EMT signature of Byers *et al*. using publically available GEO dataset GSE4824. Probes for only 48 of the EMT signature genes were contained within the array data. Therefore, these 48 genes were surveyed. GSE4824 includes samples from >75 lung cancer cell lines and was used in derivation of the EMT gene signature [23]. The gene most inversely related to *VDR* was the mesenchymal marker *ZEB1*, with a correlation coefficient of −0.385. The three genes which showed the strongest positive correlation with *VDR* were *TACSTD2*, *SH3YL1* and the epithelial marker, *CDH1* (correlation coefficients between 0.73–0.77). *TACSTD2* and *SH3YL1* appear to mark cells with a more epithelial phenotype, as their expression correlates positively with *CDH1* and negatively with *VIM* [23]. The complete ranked gene-by-gene analysis is provided in Table 2. These results are consistent with our cell line experiments and support an association between *VDR* expression and EMT phenotype in NSCLC cells.

**Table 2:** Correlation between *VDR* and EMT Signature Genes in lung cancer cell lines. The correlation between expression of *VDR* (Affymetrix probe 204254_s_at) and individual EMT signature genes in GEO dataset GSE4824 is presented.

Probe ID	Gene ID	Correlation	Probe ID	Gene ID	Correlation
212764_at	ZEB1	−0.39	210715_s_at	SPINT2	0.52
210875_s_at	ZEB1	−0.36	219121_s_at	RBM35A	0.52
201426_s_at	VIM	0.01	205977_s_at	EPHA1	0.52
208510_s_at	PPARG	0.03	37117_at	PRR5	0.54
201069_at	MMP2	0.06	205709_s_at	CDS1	0.55
207847_s_at	MUC1	0.10	220318_at	EPN3	0.57
202686_s_at	AXL	0.11	210058_at	MAPK13	0.57
218792_s_at	BSPRY	0.12	212070_at	GPR56	0.58
211732_x_at	HNMT	0.24	203453_at	SCNN1A	0.59
212298_at	NRP1	0.25	202525_at	PRSS8	0.59
202454_s_at	ERBB3	0.25	200606_at	DSP	0.60
201839_s_at	TACSTD1	0.26	205980_s_at	PRR5	0.60
204112_s_at	HNMT	0.27	213285_at	TMEM30B	0.60
201428_at	CLDN4	0.29	219476_at	LRRC54	0.61
205847_at	PRSS22	0.29	218856_at	TNFRSF21	0.62
209488_s_at	RBPMS	0.30	202489_s_at	FXYD3	0.62
35148_at	TJP3	0.30	203397_s_at	GALNT3	0.63
214702_at	FN1	0.32	221610_s_at	STAP2	0.64
202005_at	ST14	0.34	219919_s_at	SSH3	0.66
216905_s_at	ST14	0.36	203780_at	MPZL2	0.67
202790_at	CLDN7	0.38	219411_at	ELMO3	0.68
204503_at	EVPL	0.40	218677_at	S100A14	0.68
65517_at	AP1M2	0.41	203256_at	CDH3	0.69
201506_at	TGFBI	0.47	201650_at	KRT19	0.72
218186_at	RAB25	0.48	201131_s_at	CDH1	0.73
218261_at	AP1M2	0.49	204019_s_at	SH3YL1	0.74
211719_x_at	FN1	0.50	202286_s_at	TACSTD2	0.77

### 2.2. Analysis of the Effects of 1,25(OH)_2_D_3_ on EMT Related Genes and Migration of SK-LU-1 cells

**Figure 2:**
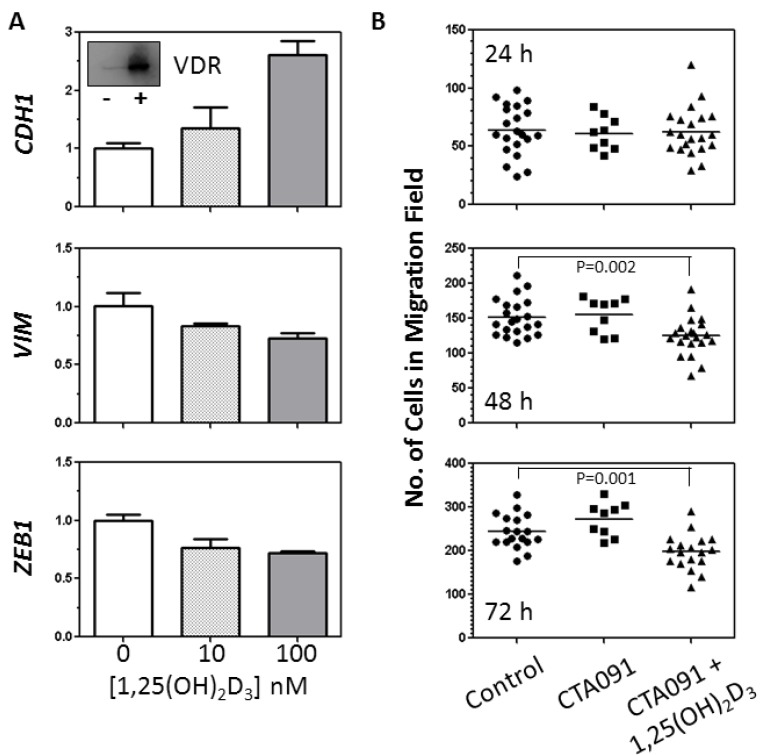
1,25(OH)_2_D_3_ supports the acquisition of an epithelial phenotype in SK-LU-1 cells and significantly decreases their migration. (**A**) SK-LU-1 cells were seeded into 6-well dishes and then treated with vehicle (controls) or 1,25(OH)_2_D_3_. Treatments were replaced every two days. After 96 h, RNA was extracted. qRT-PCR was used to measure expression of the epithelial marker *CDH1* and the mesenchymal markers *VIM* and *ZEB1*. Data are the mean ± SD for triplicate measurements within a single experiment. The expression of each gene was normalized to the level obtained for vehicle treated cells. Similar results were obtained in a second, independent experiment. Inset shows VDR protein expression [20 µg/lane for vehicle treated cells (−) and cells treated with 100 nM 1,25(OH)_2_D_3_ (+)]; (**B**) SK-LU-1 cells were seeded into ibid cell culture inserts as outlined in the Experimental Section. The next day, the inserts were removed, and cells were treated with fresh medium containing vehicle (controls), CTA091 (50 nM), or 100 nM 1,25(OH)_2_D_3_ plus CTA091 (50 nM). The number of cells that migrated into the open field at various times post-treatment (h) was determined. Migration was measured at three locations within each well, and the data from 3 independent experiments (1−3 wells/experiment) was pooled. Each data point reflects a separate measurement, and horizontal bars indicate the mean cell number. Data were analyzed for statistical significance using an unpaired t-test.

Cumulatively, the above data suggest that NSCLC cells with an epithelial gene signature have higher expression of VDR and greater sensitivity to 1,25(OH)_2_D_3_ treatment than cells with a mesenchymal phenotype. VDR/1,25(OH)_2_D_3_ signaling has been shown to influence the EMT in rat lung epithelial cells and in breast and colon cancer cells [12,14,26]. Therefore, we next sought to determine whether in NSCLC cells 1,25(OH)_2_D_3_ actively supports the epithelial phenotype or is simply correlated with it. To do this, we treated SK-LU-1 cells with vehicle or increasing concentrations of 1,25(OH)_2_D_3_. After 96h, RNA was isolated and the expression of *CDH1*, *VIM*, and *ZEB1* was measured by qRT-PCR. SK-LU-1 cells were used for these studies because they had an intermediate EMT phenotype and retained VDR expression (Figure 2A inset) and so might be susceptible to regulation by 1,25(OH)_2_D_3_. Indicative of an active role for 1,25(OH)_2_D_3_ in regulation of the EMT in SK-LU-1, treatment resulted in a 2.6-fold increase in *CDH1* expression and a modest 30%–50% decrease in expression of both *VIM* and *ZEB1* (Figure 2A).

To ascertain whether such changes in gene expression might have functional relevance, we subsequently evaluated the effect of 1,25(OH)_2_D_3_ treatment on the migration of SK-LU-1 cells. SK-LU-1 cells robustly induce expression of the vitamin D catabolizing enzyme *CYP24A1* in response to 1,25(OH)_2_D_3_ treatment (Table 1). Based on our prior work in NSCLC cells, *CYP24A1* induction was expected to result in a time-dependent decline in 1,25(OH)_2_D_3_ levels [27]. To avoid the need for periodic replenishment of 1,25(OH)_2_D_3_ and minimize disruption of the cell monolayers during the migration assays, the CYP24A1 selective inhibitor, CTA091 was added in combination with 1,25(OH)_2_D_3_. CTA091 itself had no effect on cell migration at any of the time points examined (Figure 2B). In contrast, treatment of SK-LU-1 cells with 1,25(OH)_2_D_3_ plus CTA091 for 48 h or greater resulted in significant inhibition of cell migration.

### 2.3. Analysis of the Effects of 1,25(OH)_2_D_3_ on TGFβ Induction of the EMT in VDR^high^ NSCLC cells

**Figure 3:**
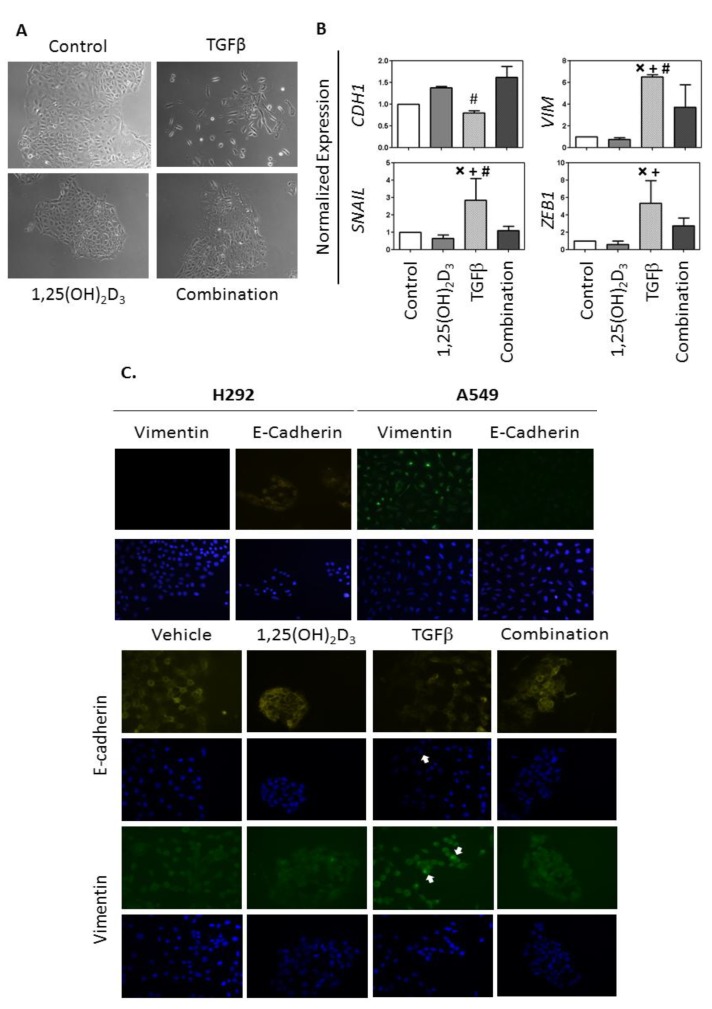
1,25(OH)_2_D_3_ opposes TGFβ induction of the EMT in HCC827 cells. HCC827 cells were seeded into 6-well plates at a density of 5 × 10^3^ cells/well. Treatments were initiated 48 h after seeding and were repeated every other day for a total of 4 treatments. The experiment was terminated 4 h after the final treatment. (**A**) Representative photographs of treated cells; (**B**) qRT-PCR was used to measure the expression of genes associated with EMT. The expression of each gene was normalized to the level obtained for control cells, which were arbitrarily assigned a value of 1.0. Data represent the mean ± SD for 3 independent experiments. The normalized data were analyzed by ANOVA with a post-hoc Tukey’s multiple comparison test (X, control *vs.* TGFβ *p* < 0.05; +,1,25(OH)_2_D_3_ *vs.* TGFβ *p* < 0.05; #, TGFβ *vs.* combination *p* < 0.05). (**C**) Cells were seeded onto glass slides and treated as outlined in (**A**). Four h after the final treatment, cells were fixed and stained with PE-conjugated E-cadherin antibodies or Alexa 488-conjugated VIM antibodies. Nuclei were visualized with DAPI. Immunofluorescence controls included untreated H292 (E-cadherin positive, VIM negative) and A549 cells (E-cadherin negative, VIM positive).

TGFβ treatment induces EMT in epithelial cells (reviewed in [28]). Therefore, as a further test of the effect of 1,25(OH)_2_D_3_ on EMT regulation in NSCLC, the ability of 1,25(OH)_2_D_3_ to oppose TGFβ induction of the EMT in HCC827 cells was determined. To do this, HCC827 cells were left untreated (controls) or were treated with 0.125 ng/mL TGFβ, 100 nM 1,25(OH)_2_D_3_, or the combination of TGFβ plus 1,25(OH)_2_D_3_ for 96 h. The effect of treatment on cell morphology was ascertained by light microscopy (Figure 3A), and the expression of *CDH1*, *VIM*, *SNAIL*, and *ZEB1* was quantified by qRT-PCR (Figure 3B). When left untreated, HCC827 cells have a cuboidal shape and form a tight monolayer. In response to TGFβ administration, the cells become spindle shaped and form loose colonies. Cells treated with the combination of TGFβ plus 1,25(OH)_2_D_3_ have a morphology more similar to controls.

With regard to gene regulation, TGFβ treatment resulted in a significant decrease in *CDH1* expression and a significant increase in expression of *VIM*, *SNAIL*, and *ZEB1* (Figure 3B). Although 1,25(OH)_2_D_3_ alone had no significant effects on gene expression, it suppressed the effects of TGFβ in HCC827 cells. Specifically, expression of *VIM* and *SNAIL* was significantly decreased in cells treated with 1,25(OH)_2_D_3_ plus TGFβ as compared to TGFβ alone (Figure 3B). Although not statistically significant, *ZEB1* expression was also 50% lower in cells treated with 1,25(OH)_2_D_3_ plus TGFβ as compared to TGFβ alone in each of three independent experiments (Figure 3B). A similar suppressive effect of 1,25(OH)_2_D_3_ on TGFβ induction of EMT was observed when the TGFβ concentration was increased to 1 µg/mL (data not shown).

To determine whether changes in RNA expression resulted in corresponding changes in protein expression, HCC827 cells were treated and then analyzed for expression of E-cadherin and VIM by immunofluorescence. Untreated H292 (E-cadherin positive, VIM negative) and A549 cells (E-cadherin low, VIM positive) were included as staining controls. Consistent with the RNA-based data, TGFβ treatment resulted in a decrease in E-cadherin expression in at least some cells (unstained cells indicated with white arrow in Figure 3C) and bright focal expression of VIM (example shown with white arrows in Figure 3C). These same bright foci were observed following VIM staining of control A549 cells but not H292 cells, indicating they are VIM specific. Conversely, 1,25(OH)_2_D_3_-treated cells displayed bright E-cadherin staining at the plasma membrane and close connectivity between cells. Cells treated with the combination of 1,25(OH)_2_D_3_ plus TGFβ had a staining pattern that was generally consistent with 1,25(OH)_2_D_3_ alone: both E-cadherin positive cell clusters and an absence of VIM bright foci were noted. We conclude from these morphological observations, gene expression profiles, and immunofluorescence data that the ability of TGFβ to induce an EMT in HCC827 cells is attenuated in the presence of 1,25(OH)_2_D_3_.

### 2.4. Discussion

Recently, a 76-gene signature was defined which distinguishes NSCLC cells based on their EMT phenotype and predicts resistance of NSCLCs to EGFR and PI3K inhibitors [23]. We build upon these findings and show that EMT phenotype (as predicted by the 76-gene EMT signature) also appears to predict resistance to vitamin D. We demonstrate that NSCLC cells which are characterized as epithelial based on the EMT signature express *VDR* and are sensitive to 1,25(OH)_2_D_3_ treatment. Conversely, NSCLC cells that are defined as having a mesechymal phenotype are relatively *VDR*-deficient and 1,25(OH)_2_D_3_-refractory. The association between vitamin D signaling capacity and EMT status led us to investigate whether vitamin D regulates the EMT in NSCLC or is simply correlated with it. We observe that the active metabolite of vitamin D, 1,25(OH)_2_D_3_, increases expression of the epithelial marker *CDH1* and decreases expression of the mesenchymal marker *VIM* in SK-LU-1 cells, where it also decreases cell migration. In HCC827 cells, 1,25(OH)_2_D_3_ opposes the ability of TGFβ to induce EMT-associated changes in cell morphology and gene expression. Cumulatively, these results support an active role for 1,25(OH)_2_D_3_ in control of the EMT in NSCLC. Our findings are consistent with prior studies showing a suppressive effect of 1,25(OH)_2_D_3_ on EMT in lung epithelial cells and breast and colon cancer cells [12,14,26].

#### 2.4.1. *VDR* Expression Is Associated with an Epithelial Phenotype and 1,25(OH)_2_D_3_ Sensitivity in NSCLC Cells

Based on the observation that *VDR* expression and vitamin D sensitivity are higher in NSCLC cells that express epithelial markers (*CDH1*, *SCNN1A*, *EPCAM*) than cells that express mesenchymal markers (*VIM*, *ZEB1*, *LIX1L*), we conclude that a relationship exists between EMT phenotype and 1,25(OH)_2_D_3_ sensitivity in NSCLC (Figure 1, Table 1). One limitation in arriving at this conclusion is that we characterized the relationship between EMT phenotype and 1,25(OH)_2_D_3_ sensitivity in a relatively small number of cell lines using only a subset of genes derived from the EMT signature. To circumvent this limitation, we examined the relationship between *VDR* and 48 genes derived from the 76 gene EMT signature using a publically available dataset containing gene expression profiles from >75 lung cancer cell lines. Using this approach, we uncovered a positive association between *VDR* and *CDH1* and a negative association between *VDR* and *ZEB1*. We believe that the results of this microarray analysis support our laboratory observations and increase the likelihood that our findings regarding EMT phenotype and 1,25(OH)_2_D_3_ sensitivity are relevant and can be generalized. We know from prior work by us and others that *VDR* expression predicts the response of NSCLC cells to 1,25(OH)_2_D_3_ treatment [24,29,30]. Thus, one implication of our current work is that an EMT signature may be useful in identifying the subset of NSCLC patients with *VDR*^high^/vitamin D responsive tumors.

#### 2.4.2. 1,25(OH)_2_D_3_ Opposes EMT Induction in NSCLC Cells

In HCC827 cells, TGFβ induces expression of *SNAIL* and *ZEB1*, master transcriptional regulators of the EMT. When TGFβ is combined with 1,25(OH)_2_D_3_, its ability to increase *SNAIL* and *ZEB1* expression is reduced. These data lead us to conclude that 1,25(OH)_2_D_3_ signaling opposes EMT induction by TGFβ. 1,25(OH)_2_D_3_ also down-regulates expression of *SNAIL* and *ZEB1* and opposes EMT induction in colon cancer cells [12,13]. The mechanistic details of the vitamin D/EMT regulatory circuit in colon cancer cells have been defined: 1,25(OH)_2_D_3_ increases expression of the histone demethylase *KDM6B/JMJD3* [12]. In turn, JMJD3 controls expression of *miR-200b* and *miR-200c*, which target *ZEB1* for degradation [13]. We are currently investigating the contribution of this mechanism towards vitamin D control of the EMT in NSCLC cells.

#### 2.4.3. EMT Signature may Identify NSCLC Patients that Benefit from 1,25(OH)_2_D_3_ Treatment

The finding of a relationship between vitamin D signaling capacity and EMT phenotype has important implications for lung cancer treatment and progression. Improvements in the treatment of advanced NSCLC have arisen from the molecular phenotyping of tumor cells and application of appropriate molecularly targeted therapies. For example, response to the EGFR tyrosine kinase inhibitor, erlotinib, is approximately 10% in an unselected population of patients with advanced NSCLC, but it is nearly 70% in those individuals whose lung tumors harbor activating mutations in *EGFR* (reviewed in [31]). To date, no gene signature has been available to identify a population of NSCLC patients that may benefit from 1,25(OH)_2_D_3_ supplementation. Based on our novel finding that a relationship exists between vitamin D sensitivity and EMT phenotype, we hypothesize that an EMT signature such as the one described by Byers et al. may prove to be clinically useful in identifying a responsive patient subset. Furthermore, our data lead us to predict that vitamin D supplementation will be effective selectively in NSCLC patients whose tumors are identified as being epithelial based on the EMT signature.

With regard to the identification of molecularly-defined lung cancer subsets that respond preferentially to vitamin D, we previously reported that NSCLC cells with activating *EGFR* mutations expressed high levels of *VDR* and were 1,25(OH)_2_D_3_ sensitive whereas NSCLC cells with oncogenic *K-ras* mutations were *VDR*-deficient and 1,25(OH)_2_D_3_-refractory [24]. When the 76-gene EMT signature was applied to 54 NSCLC cell lines, Byers *et al.* observed that all nine *EGFR* mutant cell lines included in their study had an epithelial phenotype. Conversely, *K-ras* mutations were more common in cell lines with a mesenchymal phenotype [23]. Thus, our results regarding the relationship between (a) oncogene mutations and vitamin D signaling capacity and (b) EMT status and vitamin D signaling capacity are concordant. In light of our new data, we speculate that the basis for the prior association we noted between oncogenic mutations and vitamin D sensitivity may not have resulted from a specific effect of the mutations on vitamin D signaling capacity per se. Rather, these mutations may drive the NSCLC cells into a particular biological state (*EGFR* mutation/epithelial state or *K-ras* mutation/mesenchymal state) in which vitamin D responsiveness is altered. The precise mechanism by which vitamin D signaling becomes silenced as lung cancer cells acquire a mesenchymal phenotype remains to be determined. One possibility is that the EMT transcriptional regulator SNAIL binds to the *VDR* promoter and represses its transcription [32].

## 3. Experimental

### 3.1. Cell Culture

HCC827, H23, A427, SK-LU-1, H3122, H292 and A549 cells were purchased from the American Type Culture Collection (ATCC, Manassas, VA, USA). A549 cells were cultured in BME medium supplemented with 2 mM glutamine (Life Technologies, Grand Island, NY, USA). HCC827, H23, H3122, and H292 cells were cultured in RPMI 1640 containing 2 mM glutamine (Corning, Tewksbury, MA, USA). H292 cells received additional supplementation with 1mM sodium pyruvate and 10 mM HEPES buffer. SK-LU-1 and A427 cells were cultured in EMEM containing 2 mM glutamine (ATCC). Unless otherwise specified, all media preparations contained 10% fetal bovine serum (FBS, Tissue Culture Biologicals, Tulare, CA, USA) and 100 U/mL penicillin-streptomycin. Cells were incubated at 37 °C with 5% CO_2_. All cells were periodically tested for mycoplasma and consistently found to be negative. No cells were used for experimental studies beyond 25 passages in our laboratory.

### 3.2. Reagents and Chemicals

The vitamin D metabolite, 1,25(OH)_2_D_3_, was generously provided as a 480 μM stock in absolute ethanol by Dr. Candace Johnson (Roswell Park Cancer Institute, Buffalo, NY, USA). Immediately prior to use, the stock was diluted to a final concentration of 10 or 100 nM in fresh tissue culture medium. Recombinant human TGFβ1 (R&D Systems, Inc. Minneapolis, MN, USA) was prepared as a stock of 20 ng/µL in 4 mM HCl containing 0.5% BSA. Immediately prior to use, it was diluted in fresh tissue culture media to a final concentration of 0.125 ng/mL. For studies involving TGFβ1, the treatments were replenished every two days. CTA091 was kindly provided by Cytochroma, Inc (Markham, ON, Canada). It was diluted and handled as described previously [24].

### 3.3. RNA Isolation

For EMT studies, cells were seeded in six well plates at a density of 5 × 10^3^ cells per well and were treated with either vehicle control, 1,25(OH)_2_D_3_, 0.125 ng/mL TGFβ1, or the combination of TGFβ1 and 1,25(OH)_2_D_3_. Treatments were replenished every two days for a total of four treatments. Four hours following the last treatment, cells were collected in TRI-reagent (Direct-Zol RNA Mini-Prep Kit, Zymo Research, Irvine, CA, USA) to initiate RNA extraction. RNA isolation was carried out per manufacturer’s instructions. RNA concentrations were read using a NanoDrop. All RNA had a 260/280 ratio of at least 2. Eluted RNA was stored at −80 °C until further use.

### 3.4. cDNA Synthesis 

500 ng of RNA was converted to cDNA using a High Capacity cDNA Reverse Transcription Kit, which included an RNase inhibitor (Applied Biosystems, Foster City, CA, USA). A 20 μL reaction was prepared, and cDNA synthesis was carried out following the manufacturer’s instructions.

### 3.5. Real-Time PCR

Real-time PCR reactions were prepared using the Maxima SYBR green/ROX qPCR Master Mix (Thermo Scientific, Pittsburgh, PA, USA) and run on a 7300 Real Time PCR System (Applied Biosystems). A volume of 1 μL of cDNA was added per reaction. The reactions were run at 50 °C for 2 min, 95 °C for 10 min, and then subjected to 40 cycles of 95 °C for 20 s, 56 °C for 25 s and 72 °C for 27 s. Data was collected during the 72 °C extension step. Relative gene expression was calculated using the 2^−ΔΔCt^ method. All primers were purchased from Integrated DNA Technologies. Primer sequences are as follows (F: forward; R: reverse) in Table 3.

**Table 3:** Primer Sequences.

Gene	primer
*VIM*	F: 5'-TGCCCTTAAAGGAACCAATGAGTC-3' R: 5'-ATTCACGAAGGTGACGAGCCAT-3'
*ZEB1*	F: 5'-TCCAGCCAAATGGAAATCAGGATG-3' R: 5'-CAGATTCCACACTCATGAGGTCTT-3'
*SNAIL*	F: 5'-TAGCGAGTGGTTCTTCTGCG-3' R: 5'-CTGCTGGAAGGTAAACTCTGGA-3'
*CDH1*	F: 5'-TGGACCGAGAGAGTTTCCCT-3' R: 5'-ACGACGTTAGCCTCGTTCTC-3'
*GAPDH*	F: 5'-CTCCTCTGACTTCAACAGCG-3' R: 5'-GCCAAATTCGTTGTCATACCAG-3'
*VDR*	F: 5'-ATAAGACCTACGACCCCACCTA-3' R: 5'-GGACGAGTCCATCATGTCTGAA-3'
*CYP24A1*	F: 5'-GCACAAGAGCCTCAACACCAA-3' R: 5'-AGACTGTTTGCTGTCGTTTCCA-3'
*SCNN1A*	F: 5'-GTCTCCCTCTGTCACGATGGTCA-3' R: 5'-ACCAGTATCGGCTTCGGAACCT-3'
*EPCAM*	F: 5'-GAGCGAGTGAGAACCTACTGG-3' R: 5'-ACGCGTTGTGATCTCCTTCT-3'
*LIX1L*	F: 5'-GCTTTGGGAGTTTCCAGTTTTGCC-3' R: 5'-CCCTGTATTTGGGTTGTCAGCTTC-3'

### 3.6. Clonogenic Assay

Cells were seeded in triplicate wells in complete growth medium at a density optimized for each cell line. Cells were treated with either vehicle control or 1,25(OH)_2_D_3_ every two days for 10 days. At the time of harvest, colonies were fixed by adding 2 mL of 70% methanol per well for 5 min. This step was repeated, and the colonies were then stained using 2 mL of 0.1% crystal violet for 5 min. Wells were rinsed with water and dried for 24 h prior to quantitation. Colonies were inspected microscopically, and a colony was defined as a cluster of at least 30 cells. To calculate the percent colonies remaining, the following equation was used: % colonies remaining = 100 × [number colonies for treatment group/average number colonies for control group].

### 3.7. Migration Assay

SK-LU-1 cells were trypsinized and resuspended in complete tissue culture medium to a concentration of 2 × 10^5^ cells/mL. One cell culture migration insert (ibidi, Verona, WI, USA) was placed into one well of a six-well plate. A volume of 70 μL of the cell suspension was placed into each side of the insert. The next day, the inserts were removed, and 2 mL of treatment medium was added. Treatments included vehicle control, 1,25(OH)_2_D_3_, CTA091 or the combination of 1,25(OH)_2_D_3_ plus CTA091. Pictures were taken each day from the time the inserts were removed until study termination using a Leica DMIL microscope equipped with a Leica ICC50 HD camera. Three images were taken per culture insert in the left, middle, and right viewing fields and were quantified by counting the number of cells that migrated into the open field. Each image was treated as a separate measurement.

### 3.8. Preparation of Whole Cell Extracts and Immunoblotting

Protein extraction and immunoblotting was done as described by us previously [24]. The following primary antibodies were used: mouse anti-human E-cadherin, clone 36 (BD Transduction Laboratories, San Jose, CA, USA); mouse anti-human Vimentin, clone RV202 (BD Pharmingen, San Diego, CA, USA); rat anti-VDR, clone 9A7 (Thermo Scientific, Rockford, IL, USA), and rabbit anti-actin (sc-1616-R, Santa Cruz Biotechnology, Dallas, TX, USA). Antibodies against E-cadherin, Vimentin, and VDR were used at a dilution of 1:1,000. Anti-actin antibody was used at a dilution of 1:2,000.

### 3.9. Immunofluorescence

Cells were seeded onto sterile coverslips at a density of 5 × 10^3^/well. The next day, cells were treated with vehicle, TGFβ, 100 nM 1,25(OH)_2_D_3_, or the combination of TGFβ plus 1,25(OH)_2_D_3_. Treatments were replenished every 48 h, for a total of 96 h. Four h after the final treatment, cells were washed two times with PBS at 37 °C (5 min per wash). Cells were fixed with a solution of 4% formaldehyde in PBS for 30 min at room temperature. The formaldehyde was removed, and cells were washed with PBS (as above). Fixed cells were permeabilized with 0.5% Triton-X100 solution made in PBS for 15 min and then washed three times (5 min per wash). Blocking was performed by adding a 1% w/v BSA solution (Bovine albumin, Sigma Aldrich, St. Louis, MO, USA) made in PBS. PE-conjugated anti-E-cadherin antibody (clone 36, BD Pharmingen) or Alexa Fluor 488-conjugated anti-VIM antibody (clone RV202, BD Pharmingen) were diluted in 1% BSA and exposed to the cells overnight. The next day, the cells were washed, stained with DAPI, and mounted to microscope slides (Molecular Probes, Invitrogen, Grand Island, NY, USA). Images were taken using QCapture software.

### 3.10. Microarray Analysis

Gene expression profiles of NSCLC cells along with their annotation were downloaded from NCBI’s Gene Expression Omnibus repository (GSE4824) [33]. The Epithelial-Mesenchymal Transition (EMT) gene signature was obtained from [1]. The expression values of the *VDR* gene (probe 204254_s_at) and the EMT signature genes were extracted and the correlation between *VDR* and each of the EMT signature genes was calculated and ranked. The analysis was performed using the statistical computational environment R Version 2.15.2 [34].

GSE4824 contains 164 samples, with 6 samples profiled by the Affymetrix Plus2.0 platform, 79 samples profiled by the Affymetrix U133A platform, and 79 samples profiled by the Affymetrix U133B platform. Since there are no *VDR* probes in the U133B platform, the 79 samples profiled by the Affymetrix U133B platform were discarded. The EMT gene signature contains 96 Affymetrix probes for 76 genes. Because 42 probes were not in the U133A platform, we discarded them from the analysis. Hence, our final analysis included 54 probes (from 48 unique genes) which are available in both the Affymetrix Plus2.0 and U133A platforms. 

## 4. Conclusions

Studies presented in this manuscript provide evidence that (A) a relationship exists between EMT phenotype and vitamin D sensitivity in NSCLC and that (B) 1,25(OH)_2_D_3_ actively suppresses EMT in at least some NSCLC cells. These results have two important clinical implications. First, as noted above, our work suggests that an EMT signature may be useful in identifying the subset of NSCLC patients with *VDR*^high^/vitamin D responsive tumors. In lung cancer, the EMT is associated with increased tumor cell proliferation, invasion, migration, metastasis, and chemotherapy resistance [17,18,35,36]. Thus, the second implication of our work is that by suppressing EMT, 1,25(OH)_2_D_3_ may prevent or reduce the onset of metastatic disease, may enhance response to chemotherapy, or may delay the development of resistance to conventional chemotherapy and molecularly targeted agents. The effect on EMT, combined with the documented ability of 1,25(OH)_2_D_3_ to directly suppress the growth of NSCLC cells via cell cycle inhibition [25,29], provides a reasonable explanation for the observed favorable association between vitamin D status and better outcomes in NSCLC [37,38].
